# COVID-19-Associated Pneumonia: Radiobiological Insights

**DOI:** 10.3389/fphar.2021.640040

**Published:** 2021-05-25

**Authors:** Sabine François, Carole Helissey, Sophie Cavallero, Michel Drouet, Nicolas Libert, Jean-Marc Cosset, Eric Deutsch, Lydia Meziani, Cyrus Chargari

**Affiliations:** ^1^ Department of Radiation Biological Effects, French Armed Forces Biomedical Research Institute, Brétigny-sur-Orge, France; ^2^ Clinical Unit Research, HIA Bégin, Paris, France; ^3^ Percy Army Training Hospital, Clamart, France; ^4^ Centre de Radiothérapie Charlebourg/La Défense, Groupe Amethyst, La Garenne-Colombes, France; ^5^ Department of Radiation Oncology, Gustave Roussy Comprehensive Cancer Center, Villejuif, France; ^6^ INSERM U1030, Université Paris Saclay, Le Kremlin Bicêtre, France

**Keywords:** radiation therapy, SARS-CoV-2 pneumonia, immune system, radiation-induced cancers, radiobiology

## Abstract

The evolution of SARS-CoV-2 pneumonia to acute respiratory distress syndrome is linked to a virus-induced “cytokine storm”, associated with systemic inflammation, coagulopathies, endothelial damage, thrombo-inflammation, immune system deregulation and disruption of angiotensin converting enzyme signaling pathways. To date, the most promising therapeutic approaches in COVID-19 pandemic are linked to the development of vaccines. However, the fight against COVID-19 pandemic in the short and mid-term cannot only rely on vaccines strategies, in particular given the growing proportion of more contagious and more lethal variants among exposed population (the English, South African and Brazilian variants). As long as collective immunity is still not acquired, some patients will have severe forms of the disease. Therapeutic perspectives also rely on the implementation of strategies for the prevention of secondary complications resulting from vascular endothelial damage and from immune system deregulation, which contributes to acute respiratory distress and potentially to long term irreversible tissue damage. While the anti-inflammatory effects of low dose irradiation have been exploited for a long time in the clinics, few recent physiopathological and experimental data suggested the possibility to modulate the inflammatory storm related to COVID-19 pulmonary infection by exposing patients to ionizing radiation at very low doses. Despite level of evidence is only preliminary, these preclinical findings open therapeutic perspectives and are discussed in this article.

## The Context

First cases of the new coronavirus (COVID-19) were detected in Wuhan in December 2019 (Zhu et al., 2020). On January 30, 2020, the World Health Organization (WHO) officially declared the COVID-19 epidemic as a public health emergency of international concern. A year has passed and despite unprecedented health measures, the number of deaths linked to this virus is now approximately 2,412,000 worldwide, including more than 305.700, in Europe and 117,160 and in United Kingdom. COVID-19 is a potentially serious illness caused by the Coronavirus 2 of Severe Acute Respiratory Syndrome (SARS-CoV-2) (https://fr.statista.com/statistiques/1101324/morts-coronavirus-monde/).

Coronaviruses represent a large family of viruses that can cause a wide range of illnesses in humans, ranging from common cold symptoms to life-threatening SARS (Yin and Wunderink, 2018; Malik, 2020). SARS-CoV-2 belongs to the beta-coronavirus subfamily ß-CoV and internalizes the body via the respiratory tract or through the mucosa (e.g., eyes). The virus may spread via saliva, respiratory secretions or droplets, which can be expelled into the ambient air by an infected person through coughs and/or sneezes and may remain suspended in the air for several hours. Its spread in the population is mainly through close contacts or aerosolization of viral particles into insufficiently ventilated indoor spaces (Anderson et al., 2020). When SARS-CoV-2 infects the respiratory tract, it causes pneumonia (often pauci-symptomatic) and may evolve to acute respiratory distress syndrome (ARDS) in about 15% of cases (Ragab et al., 2020).

Mortality in COVID-19 patients is linked to a virus-induced “cytokine storm” (Hu et al., 2020; Song et al., 2020). This is a continuous mechanism involving hyper-activation of immune cells, including lymphocytes and macrophages producing large amounts of pro-inflammatory cytokines such as IL-1, IL-6, IL-18, IFN-γ, and TNF- a leading to worsening of ARDS with the appearance of generalized tissue damage, potentially leading to multi-organ failure and patient death (Fara et al., 2020). Since the start of the pandemics, other clinical manifestations concomitant with pneumonia following viral infection have been described. Those include coagulopathies (activation of coagulation) and cardiac dysfunctions contributing to mortality, and even being the main cause of death in some patients who develop arrhythmias, acute coronary syndromes and venous thromboembolic events (Middeldorp et al., 2020; Nishiga et al., 2020; Ribes et al., 2020). The pathophysiology of COVID-19 cardiac disease also leads to direct myocardial lesions consecutive to viral-related cardiomyocyte damage, and is potentiated by the consequences of systemic inflammation that is a major and common mechanism responsible for cardiac damage (Bansal, 2020).

Severe forms of COVID-19 are preferentially observed in the elderly population, in people with underlying health problems such as diabetes and in those with deficit in their immune system (Shahid et al., 2020). In severe cases of COVID-19, damages can spread beyond the lungs to other organs, including the heart, kidneys, liver, brain, eyes, gastrointestinal tract, skin, and bone marrow with its stem cell compartments and hematopoietic progenitors, (Cipriano et al., 2020; Gupta et al., 2020; Khaled and Hafez, 2020). The presence of viral RNA is detected post-mortem in the endothelial cells of many organs, revealing endothelitis (Jung et al., 2020; Varga et al., 2020). Endothelial damage and thrombo-inflammation, immune system deregulation and disruption of angiotensin converting enzyme (ACE2) signaling pathways could contribute to the onset of these extra-pulmonary manifestations of COVID-19. The expression of ACE2 in the tissues facilitates the penetration of SARS-CoV-2, by enabling the virus to propagate to the cells of many organs, thereby decreasing the expression of this protein within the infected cells themselves and increasing expression of angiotensin II (Ang II) (Kuba et al., 2005; Banu et al., 2020; Bourgonje et al., 2020). Furthermore, ACE-2 expression is found in endothelial cells, smooth muscle cells and perivascular pericytes of the vast majority of organs. SARS-CoV-2, once present in the circulation, can therefore easily spread to other parts of the body (Huertas et al., 2020). ACE2 has anti-inflammatory and anti-fibrotic properties through its function of conversion of angiotensin (Ang–II) into Ang (1–7), and its decreased expression caused by the virus promotes disruption of the immune system and contribute to the development of tissue fibrosis. Combined with the activation of macrophages, such impact on ACE2 could be involved in the development of COVID-19-related fibrosis (He et al., 2006; Meng et al., 2014; Patel et al., 2016; Rodrigues Prestes et al., 2017; Smigiel and Parks, 2018; Banu et al., 2020; Pagliaro, 2020). To date, the most promising therapeutic approaches in COVID-9 pandemic are linked to the development of vaccines. However, the fight against COVID-19 pandemic in the short and mid-term cannot only rely on vaccines strategies, in particular given the growing proportion of more contagious and more lethal variants among exposed population. As long as collective immunity is still not acquired, some patients will have severe forms of the disease. Therapeutic perspectives also rely on the implementation of strategies for the prevention of secondary complications resulting from vascular endothelial damage and from immune system deregulation, which contributes to acute respiratory distress and potentially to long-term tissue fibrosis.

The C5a complement factor and its receptor (C5aR1) have key roles in the initiation and maintenance of inflammatory processes by recruiting neutrophils and monocytes, contributing to the pathophysiology of COVID-19 related acute respiratory distress syndrome. The levels of soluble C5a are increased in proportion to the severity of COVID19 infection. In animal models, inhibition of anti-C5aR1 axis prevented the C5a-mediated recruitment and activation of human myeloid cells in damaged lungs. These data open pharmacological perspectives for the modulation of COVID-19 related inflammation(Carvelli et al., 2020).

Several recent physiopathological and experimental data suggest the possibility to modulate the inflammatory storm related to COVID-19 pulmonary fsfsfs by exposing patients to ionizing radiation at very low doses. Despite level of evidence is only emerging, these preclinical findings open therapeutic perspectives and are discussed in this article.

## Pathophysiological Mechanisms of the Respiratory Complications of COVID-19

The diagnosis of ARDS is conventionally based on well-defined parameters using the Berlin criteria, the oxygenation index and the Murray/lung Injury Score used by intensive care physicians to define the clinical, ventilatory, gasometric parameters (analysis of blood gas) and radiological criteria to establish the diagnosis of this serious pulmonary syndrome and to adapt the ventilatory management as well as possible (ARDS Definition Task Force et al., 2012; Huber et al., 2020). Respiratory physiology in patients developing COVID-19 differs from the ”conventional” acute respiratory distress syndrome (ARDS) (Gattinoni et al., 2020). Indeed, there is an aberrant activation of the inflammatory system and coagulation processes, and this pattern is somewhat characteristic of the “immuno-thrombostic” process observed in COVID-19 pneumonia (Nakazawa and Ishizu, 2020). The classical ARDS pneumonitis seen in patients infected with SARS-CoV-2 is characterized by a decrease in lung distension capability. Damages to lung tissue strongly affect the level of ventilation capability. Many unventilated areas are filled with fluid (alveolar edema) and cells. The alveolar air is replaced by a pathological product, which leads to abnormal opacities (alveolar condensations), as seen on computed tomography scans.

**FIGURE 1:**
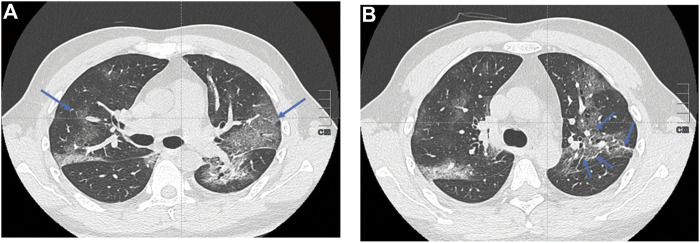
Chest computed tomography images of patients with COVID-19 pneumonia **(A)** shows Ground-glass opacities (blue arrows) **(B)** shows confluent crazy-paving pattern and consolidation opacities: secondary appearance of intralobular reticulations (blue arrows).

**FIGURE 2:**
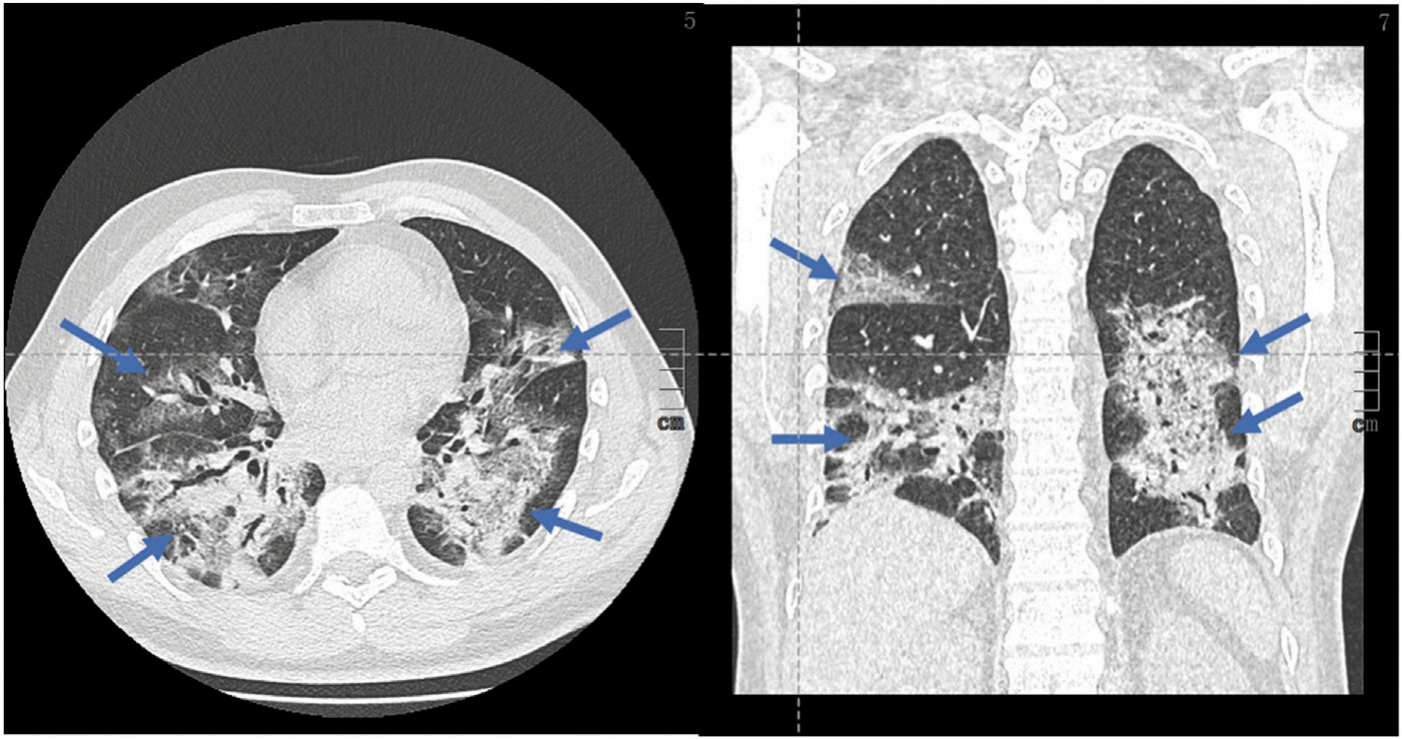
Chest computed tomography images of patients with COVID-19 pneumonia: shows extensive abnormalities and a proportion of pulmonary condensation (blue arrows) vs. higher Ground-glass, with possible progression to pulmonary fibrosis.

Chest scans are indicated to guide the management and monitoring of pulmonary symptoms in a patient with COVID-19. In addition to its use for early diagnosis, the chest scan has a prognostic role, making it possible to visually assess the extent of pulmonary lesions and monitor over time. The abnormalities observed on the CT scan are correlated to severity of clinical symptoms (Wong et al., 2003). Although radiological changes observed in the context of SARS-CoV-2 infection are not specific, those are indicative of the diagnosis in the current epidemic context. The most reported CT abnormalities are as follows: ground-glass opacities, multifocal, bilateral, and asymmetrical, with preferentially subpleural localization predominant in the basal and posterior area. The presence of bronchiolar micronodules, mediastinal lymphadenopathy and pleural effusions is also suggestive. All those characteristics may be found in pulmonary bacterial infections. At a later stage, the radiological aspects evolves toward a “crazy paving” aspect, with appearance of intralobular reticulations (peak around the 10th day) and linear condensations can be observed (Figure 1A) (ARDS Definition Task Force et al., 2012; Smigiel & Parks, 2018; Huber et al., 2020). In most severeforms of COVID19 pneumonia, CT scan shows extensive abnormalities and a higher proportion of pulmonary condensation vs. ground-glass opacities (Figure 1B). With time, weak regression of the abnormalities can be observed, often associated with so-called late fibrous sequelae (Figure 2).

Despite an unprecedented investment to look at therapeutic strategies, there is currently no effective treatment for COVID-19 infection. Most potential treatments have been evaluated in populations with significant heterogeneity and various levels of symptoms severity. Several existing antiviral treatments are being tested: remdesivir, combination lopinavir/ritonavir, combination lopinavir/ritonavir/interferon beta or even hydroxychloroquine. Remdesivir did not show effect in patients presenting with severe form of the disease, as assessed per mortality probability at day 28 (Beigel et al., 2020a; Wang et al., 2020). It nevertheless has a possible beneficial effect in non-ventilated patients (Beigel et al., 2020b). Hydroxychloroquine has shown no benefit in large clinical trials (RECOVERY Collaborative Group et al., 2020b). It also exhibits significant side effects. Lopinavir/ritonavir was unsuccessful (RECOVERY Collaborative Group et al., 2020a; Cao et al., 2020). Modulation of the immune response by specific blockade of an interleukin was not effective after initially raising high expectations. Patients who received tocilizumab had fewer serious infections than patients who received placebo. In the RECOVERY trial, tocilizumab reduced death from 33 to 29%. It also reduced the chance of progressing to invasive mechanical ventilation or death from 38 to 33%. (Stone et al., 2020; RECOVERY Collaborative Group, 2021). Plasma from convalescent patients has not shown an effect in the general population (Simonovich et al., 2020). It could nevertheless be effective in patients not developing an immune response. Monoclonal antibodies targeted against the spike protein of SARS-Cov-2 (casirivimab and imdevimab) (Baum et al., 2020) have just been authorized by the FDA for patients with mild to moderate symptoms of COVID-19. In combination, monoclonal antibodies seem to reduce the probability of hospitalization or needing urgent cares. Those however did not improve the prognosis in hospitalized patients and may even make ventilated patients worse. The only specific treatment which demonstrated a decrease in mortality is corticosteroid as an anti-inflammatory therapy, dexamethasone at a dose of 6 mg/day with a modest decrease from 25.7 to 22.9% (RECOVERY Collaborative Group et al., 2020b). The disappointing results of specific therapies underline the importance of symptomatic treatment and routine supportive care, such as adapted oxygen therapy and prophylaxis of thromboembolic disease (which frequently complicates severe cases) in combination with nonspecific treatments of organs failure (Helms et al., 2020).

The long-term respiratory sequelae of COVD-19 are also a significant clinical concern. Based on data from 2003 SARS-CoV, showing that 35–60% of survivors developed pulmonary fibrosis with reduced lung function, it can be expected that at the end of this pandemic, a high number of patients surviving severe cases of Covid-19 will be severely affected by persistent respiratory complications. The true incidence of such late fibrosis in the COVID-19 context is however still uncertain (Ronald). After ARDS following SARS-CoV-2 infection, there is a progressive accumulation of the extracellular matrix potentially leading to respiratory failure. Anatomopathological examinations carried out on patients who died of COVID-19 revealed the presence of numerous lesions of alveolar epithelial cells, the formation of hyaline membranes, type II pneumocyte hyperplasia, fibroblastic proliferation with a matrix important extracellular and fibrin deposits in alveolar spaces (Carver et al., 2007; Raghu et al., 2011; Tian et al., 2020). The mechanistic phenomenon underlying the onset of lung fibrosis following COVID-19 is poorly understood, but may involve the continued presence of the immune response causing deregulation of tissue repair. The magnitude of the cytokine storm, and severity of cell alterations within the alveolar tissue, may over time accelerate the development of fibrosis in a diffuse manner across both lungs. Lung transplants have been performed to treat patients presenting with acute respiratory failure following a COVID-19 infection. Pathological examination reveals that the virus may cause an almost complete destruction of both lungs (Hu et al., 2020). Lung transplantation could be an effective curative treatment for terminal lung diseases. However, we must remain cautious about this therapeutic possibility, because the recovery of a lung transplant patient is long and very uncertain, and access to lung transplants is highly limited worldwide (Roux et al., 2019).

## Rationale for Low Dose Irradiation in the Inflammatory Context

As pointed out by Edward J Calabrese and Gaurav Dhawan, during the first half of the 20th century, radiation therapy was used a long time ago to treat pneumonia. Fifteen studies grouping together around 700 cases of pneumonia of bacterial origin (lobar and bronchopneumonia), including those unresponsive to treatment with sulfonamides, and described as being interstitial and atypical were treated effectively with low doses of X-rays, showing a decrease in clinical symptoms, and a lowering of mortality rates (Calabrese and Dhawan, 2013). Low doses of irradiations were also used for skin or articular inflammatory diseases, with most frequently high efficacy. Low doses of irradiation have been proposed as an effective treatment option in various benign inflammatory pathologies, including osteoarthritis, keloids scares, eczema, lymphatic fistulas, age-related macular degeneration, sialorrhea and suppurative hydradenitis (chronic inflammatory skin disease) (Torres Royo et al., 2020). This approach showed a beneficial effect on autoimmune diseases such as arthritis and encephalomyelitis (chronic fatigue syndrome) (Tsukimoto et al., 2008; Nakatsukasa et al., 2010). Preclinical studies on diabetes have demonstrated an antioxidant effect of low doses of irradiation (Wang et al., 2008). These clinical and preclinical investigations provided an increasing level of evidence of the effects of low doses of irradiation, with an anti-inflammatory, anti-oxidant and anti-proliferative potential, associated with high efficacy in reducing clinical symptoms in some inflammatory pathologies.

However, the empiric beneficial effect of low doses of irradiation has been debated for over 50 years, in part because of the poor knowledge on the underlying mechanistic in the context of major concerns in terms of potential radiation-induced cancers (Jaworowski, 2010). Indeed, there is a significant risk of radiation-induced cancers among survivors from a therapeutic irradiation, and epidemiological data clearly documented an increased risk for second neoplasms in cancer survivors (Chargari et al., 2016; Chargari et al., 2020). The risk is the highest among youngest patients, and seems to be organ-dependent (highest risk for the breast and the thyroid). The question of a dose threshold for this risk, as well as the uncertainties on the shape of dose/response curve, is still unsolved. Those parameters have a major impact in the risk estimate. Anyway, the potentially carcinogenic effects of low doses of irradiation have led to almost abandon this approach to treat inflammatory diseases, and this trend was obviously accelerated by the increasing availability of highly effective non-steroidal or steroidal drugs. Scarce indications for noncancerous diseases do persist however, such as treatment of refractory keloid scares (with high efficacy and low morbidity). In Germany, approximately 50.000 patients are still referred and treated by radiotherapy for non-malignant disorders, including painful degenerative skeletal disorders, hyperproliferative disorders and symptomatic functional disorders(Seegenschmiedt et al., 2015). It should be highlighted that systemic anti-inflammatory therapies also present undesirable effects (severe bacterial complication, in particular in the case of pulmonary infection, digestive disorders such as gastritis or digestive ulcer complicated by hemorrhage, renal damage such as renal failure, necrotizing fasciitis) and a considerable number of patients do not respond correctly (Aronoff and Bloch, 2003; Rödel et al., 2007; Arenas et al., 2012; Legras et al., 2009; Arenas et al., 2012; Le Bourgeois et al., 2016; Basille et al., 2017; Voiriot et al., 2019; Point AINS.,).

**FIGURE 3:**
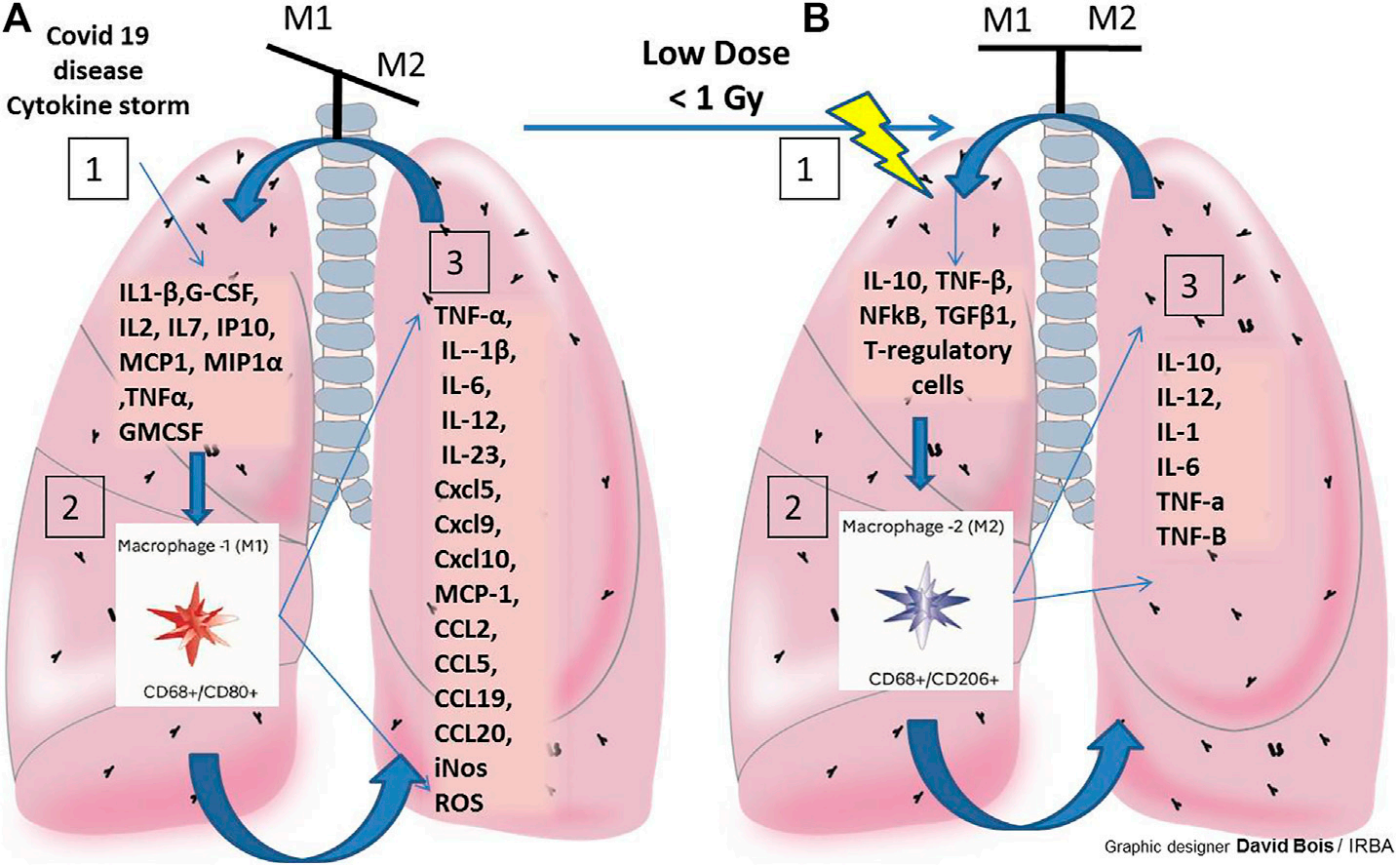
Beneficial effect of low-dose irradiation to reprogram macrophages in anti-inflammatory M2 promoting tissue repair or slowing the progression of severe lung damage induced by covid 19 disease. Balance of M1/M2 macrophage is necessary to achieve proper tissue repair. Hyperinflammation and the severity of the lesions alter this balance (illustrated above each of the lungs **A** and **B**). **(A)**: Illustrations and details of M1 macrophage stimulation in COVID19 in the lung and their pro-inflammatory potential with very little macrophage reprogramming into anti-inflammatory M2. Depending on severity and duration inflammation (M1 persistent activation) this leads to severe lung damage by covid 19 disease. **(B)**: To generate an anti-inflammatory environment: stimulate the polarization of the M2 macrophages with low dose radiotherapy (RT). Macrophages also switch to an anti-inflammatory (M2) phenotype, leading to a wound healing phase: Maintains M1/M2 balance or slowing the progression of lung damage induced by covid 19 disease. 1/2/3 represent the 3 steps generated in case A, an M1 macrophage phenotype within the lungs of covid 19 patients, in case B, step 1 (effects of low doses of RT in lung, with ↘ NO, ROS, ↘leukocytes/endothelial cells adhesion and ↗IL-10, TNF-β, NFkB, TGF 1, AP-1 et T-regulatory cells), step 2 (stimulation of the polarization of M2 macrophages in this environment post-low dose RT) and step 3: the secretion products of M2 promoting an anti-inflammatory environment.

An increasing number of preclinical investigations have been carried out to better understand the underlying anti-inflammatory effects of low doses of irradiation. In the light of recent radiobiological data, the putative mechanisms for the anti-inflammatory effects of low-dose irradiation are now well understood. Those include the following patterns: increased heme oxygenase, increased anti-inflammatory cytokines - interleukin-10 (IL-10), increased tumor necrosis factor -beta (TNF-β), activation of several transcription factors, such as nuclear factor kappa beta (NFkB) and protein-1 (AP-1), apoptosis promotion, transforming growth factor - beta 1 (TGFβ1) activation, and stimulation of the activity of regulatory T cells (Dhawan et al., 2020; Genard et al., 2017). As reviewed by Arenas and colleagues, the anti-inflammatory effects of low dose irradiation can also be explained by a decreased adhesion of polymorphonuclear cells to endothelial cells, decreased expression of adhesion molecules, such as selectins, ICAM, VCAM). Doses <0.7 Gy may modulate the expression of adhesion molecules and the production of cytokines, decreasing leukocytes/endothelial cells adherence. Other authors have reported a decrease in NO and ROS, and increased activation of NF-kB, and increase activator protein 1 (Ap-1) activity(Arenas et al., 2012). Doses of approximately 0.5 Gy can modify the immune microenvironment and exert an anti-inflammatory effect, by causing macrophage polarization toward anti-inflammatory macrophages (Lara et al., 2020). This anti-inflammatory effect of the low doses of irradiation was recently demonstrated in human lung macrophages (*Ex vivo*) and in a preclinical study, using a viral pneumonia model (influenza A PR8 virus (H1N1). Authors showed that low doses of irradiation decreased both lung damages and inflammation and had no effect on viral expansion (Meziani, 2020). These anti-inflammatory effects of low dose irradiation are attractive to mitigate the covid-19 related cytokine storms, though only few preclinical data tested this approach in animal models of viral pneumonia. Beneficial effect of low-dose irradiation to reprogram macrophages in anti-inflammatory M2 promoting tissue repair or slowing the progression of lung damage induced by covid 19 disease is detailed and illustrated in Figure 3. A recent review of radiobiological data published in 1937–1973 identified 6 studies evaluating post inoculation radiation exposure in animal models; the results were heterogeneous, with one study showing a significant increase in mortality and another showing a significant decrease associated with radiation exposure. No significant change was found in the four remaining studies. These historical preclinical results do not provide support for efficacy of post infection radiation exposure, but the added value of such old reports to the current applicability of low dose radiotherapy is uncertain (Little et al., 2020).

In spite of these limitations, several prospective trials are currently being carried out in the context of the COVID-19 pandemic, encouraged by the lack of effective alternative and the high mortality probability in most severe cases of COVID-19 pneumonia (Cosset et al., 2020; Wilson et al., 2020). In addition, the probability that such doses would result in any deterministic toxicity to healthy tissue is very low (Hanekamp et al., 2020). Most often, these studies are designed to assess the possibility to reduce the need for non-invasive or invasive ventilation by administering a very low dose of X-rays in cases of severe lung infection. To date, nine clinical studies are underway worldwide, including 3 in Spain (UTLTRA-COVID, LOWRAD-COV19), 1 study in Italy (COLOR-19) and the PREVENT study in the United States ((ongoing studies: NCT04380818, NCT04572412, NCT04534790, NCT04394182, NCT0CT044, NCT04393948, NCT04466683) (PREVENT). Preliminary results are encouraging. A clinical trial involving 5 patients over the age of 60 and hospitalized for oxygen therapy showed that a single fraction of 0.5 Gy over the entire lungs, in combination with the standard treatment then proposed, was followed by a clinical improvement in 4/5 patients (Ameri et al., 2020). In another pilot study for which only interim analysis on Day 7 is available, 5 patients with a median age of 90 years were irradiated at low doses and among them 4/5 presented a significant clinical and radiological improvement, including 3 patients within 24 h. No acute toxicity was observed and of importance, no worsening of the cytokine storm was observed in 4 of the 5 patients. As highlighted by the authors themselves, further evaluation to determine additional safety and efficacy among patients with COVID‐19 pneumonia is mandatory (Hess et al., 2020). Recently, Sanmamed et al. published a preliminary report of a prospective single arm phase I-II clinical trial enrolling patients ≥50 years-old COVID-19 positive, at phase II or III with lung involvement at imaging study and oxygen requirement. Patients were exposed to 100 cGy to total lungs in a single fraction. Among nine patients included, authors observed statistically significant changes in the disease extension score and improvement of SatO2/FiO2 index 72 h and 1 week after irradiation. In parallel, they observed that LDH decreased significantly one week after RT compared with baseline. Two patients had grade 2 lymphopenia after RT and another worsened from grade 3 to grade 4. Overall, the median number of days of hospitalization was 59 days (range 26–151). After RT the median number of days in hospital was “only” 13 days (4–77). With a median follow-up after RT of 112 days, seven patients were discharged and two patients died, one due to sepsis and the other with severe baseline chronic obstructive pulmonary disease from COVID-19 pneumonia (Sanmamed et al., 2020). These results are quite encouraging, but still those are preliminary data deserving to be validated in larger-scale trials assessing the value of low-dose pulmonary irradiation in this situation with a comparative arm. Such approach could potentially improve the quality of life of post-COVID19 patients, reduce the number of deaths and reduce patients stay in intensive care (Martin, 2003; Haas et al., 2018). In addition, the duration in intensive care is not without side effects for patients who are ventilated and immobilized by sedation over a long period. Such approach, based on the anti-inflammatory properties of low dose radiation therapy, should however be extremely cautiously tested, prioritizing the patients who have the lowest risk for second cancers (elderly population) and for whom no effective treatment is available. Indeed, trials testing low dose irradiation have to take into account the theoretical risk of radiation-induced cancer, and the paucity of supportive preclinical data to treat COVID-19 pneumonia was highlighted (Chargari et al., 2016; Haas et al., 2018; Kirsch et al., 2020). Furthermore, one cannot preclude that irradiation would exacerbate an active COVID-19 infection though an increase in the cytokine storm or lead to cardiovascular morbidity. The use of low dose radiotherapy for COVID-19 pneumonia cannot be recommended outside a clinical trial. In addition, this approach should be particularly cautious in young patients (<50–60 years) -who have in most of the cases a good prognosis of their COVID-19 respiratory disease - in particular because the mammary gland and thyroid are highly sensitive to the carcinogenic effects of ionizing radiation, though the effect of such low doses remains uncertain. The risk of second cancer must be put into perspective in the context of elderly patients, frequently ineligible for invasive resuscitation or treatment with interleukin-6 inhibitors, for whom the problem of radiation-induced cancers possibly occurring 10–20 years after irradiation is not a priority concern. Thus, it is estimated that a patient who receives low-dose pulmonary radiotherapy for the treatment of COVID-19 at an age of 80 has a theoretical risk of radiation-induced cancer of less than 1% (Chargari et al., 2016; Cosset et al., 2016).

## Conclusion

Although numerous data show that low dose radiotherapy may have anti-inflammatory properties, the evidence supporting the use of low dose radiotherapy to treat COVID-19 infection remains preliminary. This approach could potentially have a favorable cost/effectiveness ratio, for a subgroup of COVID-19 patients for whom there is most often no therapeutic alternative and in a context of lack of access to resuscitation platforms (García-Hernández et al., 2020). A prerequisite for achieving successful development of this experimental treatment is to more accurately identify what population could get benefit, if any, from this treatment, and to better determine the optimal timing/dose/fractionation to achieve the best therapeutic index with satisfactory safety profile. The superiority of low dose radiotherapy over more conventional systemic anti-inflammatory (e.g., steroids) remains undemonstrated, and only a well-designed randomized clinical trial will provide the evidence of a benefit (if any) of low dose radiotherapy in this context. A step by step process is required, from early phase trials to larger randomized studies, to ensure that the beneficial effect of low dose radiotherapy is superior to its potential side effects.
